# Supplementary material for: Endotaxial stabilization of 2D charge density waves with long-range order
Source: Nat Commun. 2024 Feb 15;15:1403. doi: 10.1038/s41467-024-45711-3 (PMC10869719; doi:10.1038/s41467-024-45711-3)
Supplement: Supplementary file 1 — Supplementary Information [file 41467_2024_45711_MOESM1_ESM.pdf]

Supplementary Information for

Endotaxial Stabilization of 2D Charge Density Waves with Long-range Order

Suk Hyun Sung, Nishkarsh Agarwal, Ismail El Baggari, Patrick Kezer, Yin Min Goh, Noah Schnitzer, Jeremy M. Shen, Tony Chiang, Yu Liu, Wenjian Lu, Yuping Sun, Lena F. Kourkoutis, John T. Heron, Kai Sun, and Robert Hovden

Contents

Supplementary Notes

Supplementary Note 1 Incommensurate CDWs and Associated PLDs 2
Supplementary Note 2 Removing CDW/PLD Diffraction Anisotropy 3
Supplementary Note 3 Cross-sectional HAADF-STEM of Endotaxial Polytype Heterostructures 4
Supplementary Note 4 Correlation Length Estimation of oIC-CDWs 5
Supplementary Note 5 Resistance Anisotropy in Pristine and Endotaxial TaS_2 6
Supplementary Note 6 Electronic Device Overview 7
Supplementary Note 7 Thermal Evolution of oIC-CDW Superlattice Peaks 8
Supplementary Note 8 Simulated Hexatic Melting of Charge Lattice and Associated Lattice Distortions 9
Supplementary Note 9 Comparison of Charge Lattice Model and Experimental Diffraction Patterns 10

Supplementary Note 1   Incommensurate CDWs and Associated PLDs

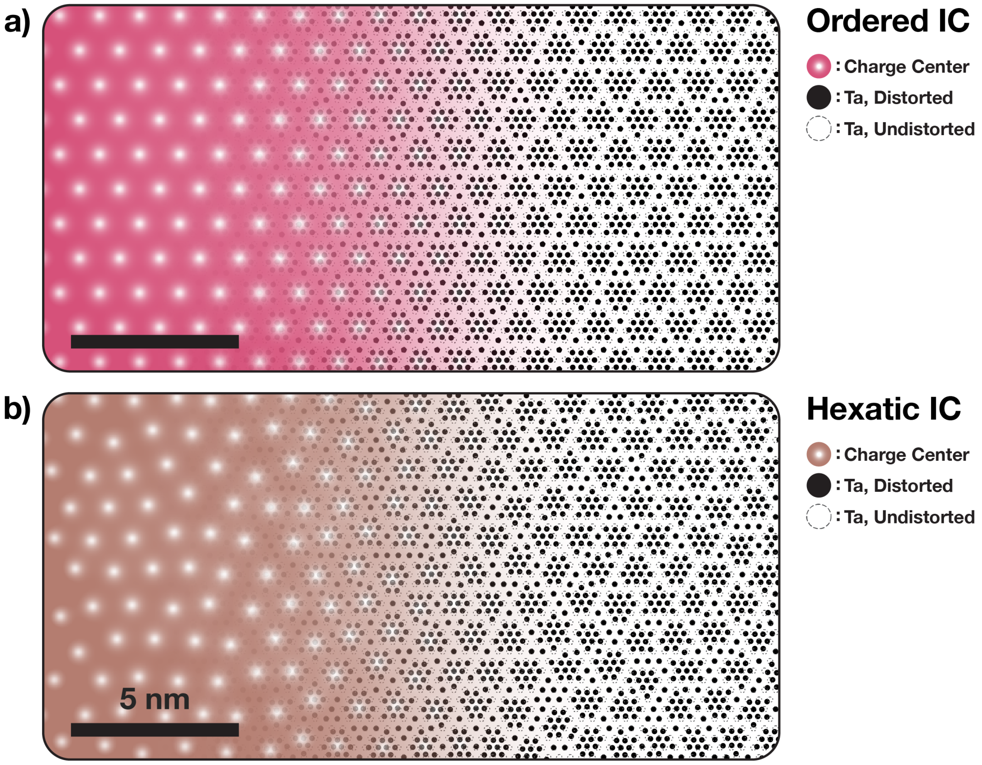

Supplementary Figure 1 | Incommensurate Charge Density Waves and Associated Periodic Lattice Distortions. a, b) Schematic representation of ordered IC-CDW and hexatic IC-CDW, respectively. White spots represents charge centers. Charge centers attract near by Ta nuclei (gray, dotted) and form local cluster of atoms (black, solid).

Supplementary Note 2 Removing CDW/PLD Diffraction Anisotropy

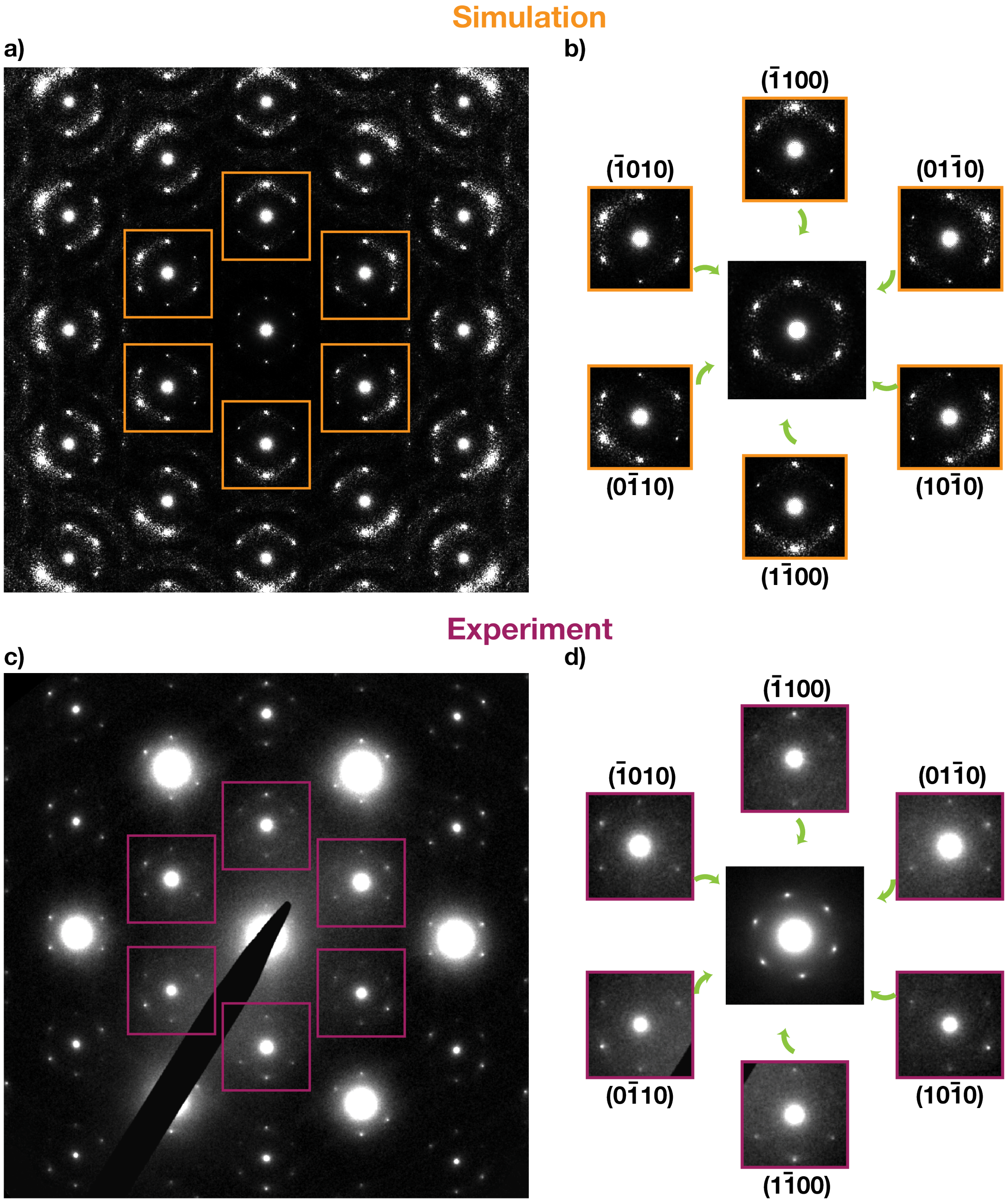

Supplementary Figure 2 | Removing CDW/PLD Diffraction Anisotropy. CDWs and PLDs manifest as satellite peaks in electron diffraction. Specifically, these superlattice peaks are anisotropically distributed around each Bragg peak [1–5]. This is clearly demonstrated in simulated (a) and experimental (c) diffraction pattern. b, d) For quantitative analysis, averaging six first order Bragg peaks evens out the anisotropy and enhances the signal to noise ratio.

Supplementary Note 3 Cross-sectional HAADF-STEM of Endotaxial Polytype Heterostructures

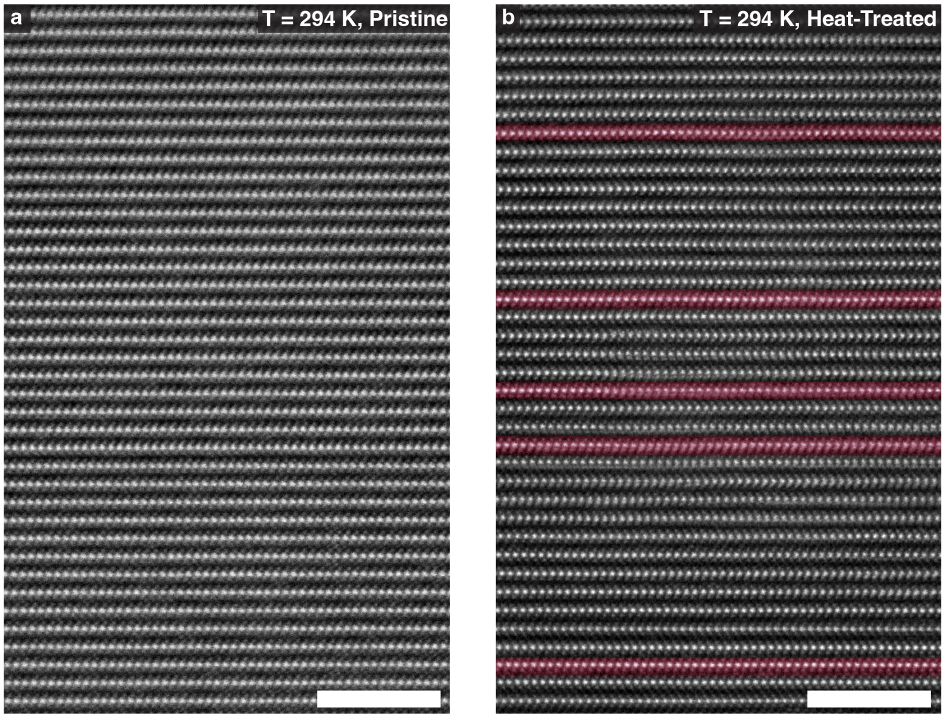

Supplementary Figure 3 | Cross-sectional HAADF-STEM of Endotaxial Polytype Heterostructures. a) $[10\bar{1}0]$ cross-sectional HAADF-STEM of $TaS_xSe_{2\text{-}x}$ of a) pristine, 1T and b) heat-treated polytypic heterostructure. b) Oc-layers are marked red. a) Every layer of 1T-$TaS_xSe_{2\text{-}x}$ is initially octahedrally coordinated as expected. b) Heat-treated sample shows Oc to Pr polytype conversion in some of layers. Monolayers of Oc-TaS_2 are shielded in between slabs of Pr-layers. Scale bar is 4 nm. A selenium doped sample was imaged to enhance chalcogen visibility and determine coordination.

Supplementary Note 4   Correlation Length Estimation for oIC-CDWs

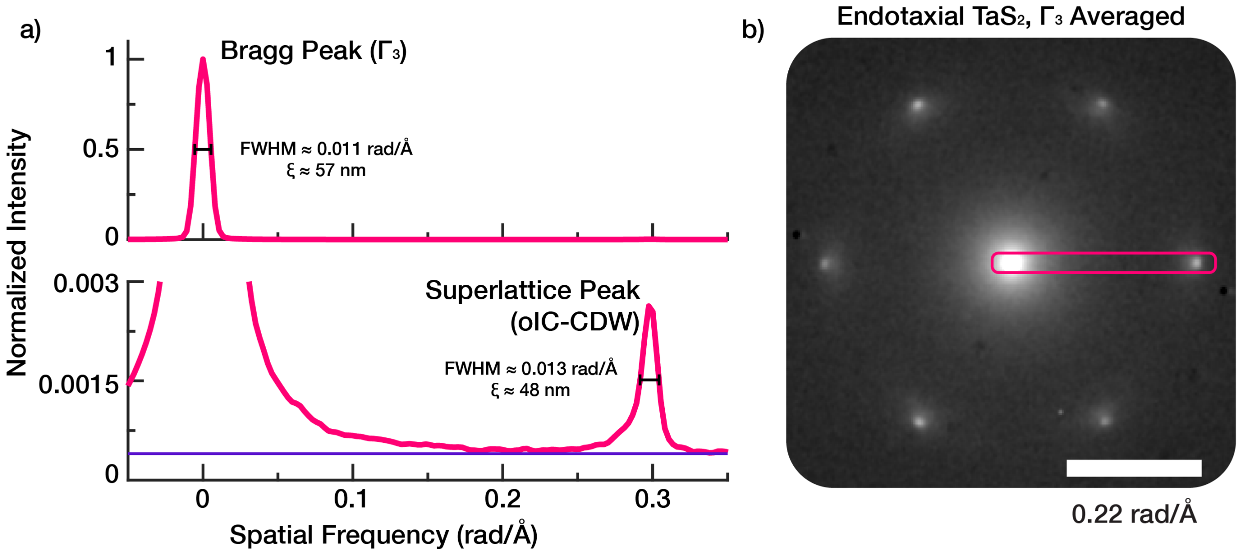

Supplementary Figure 4 | Correlation Length Estimation for oIC-CDWs in Endotaxial Polytype Heterostructures. a) SAED line-profile of thermally treated TaS_2 specimen in oIC-CDW phase. Correlation length inversely proportional to the peak width (FWHM, full width at half maximum); Correlation length $\propto \xi; \xi = \frac{2\pi}{\mathrm{FWHM}}$. Here is best to compare the CDW peak width relative to the Bragg peaks under the same imaging conditions since diffraction peak widths are determined by the crystal/ CDW order, size of the diffracting region, and the microscope optics. b) Averaged third order $\{20\bar{2}0\}$ Bragg peaks with superlattice peaks surrounding it. 6 Bragg peaks were averaged to remove anisotropy and increase signal-to-noise ratio. Magenta box denotes region where line profile (a) was taken from.

Supplementary Note 5   Resistance Anisotropy in Pristine and Endotaxial TaS_2

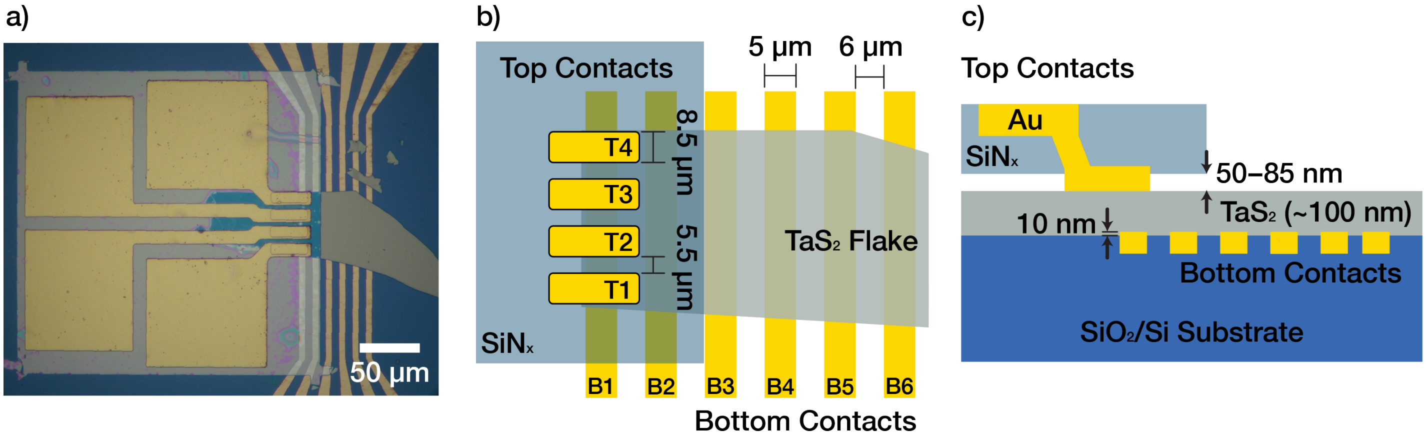

Supplementary Figure 5 | Resistance Anisotropy Measurements in Pristine and Endotaxial TaS_2. a) Optical overview of device for measuring resistance anisotropy of TaS_2 flake. Device includes 10 gold electrodes (4 on top, 6 at bottom) as well as SiN_x membrane that prevents top electrodes from side-wall conduction. Schematic diagram of the TaS_2 device from top-down (b) and cross-sectional (c) view. Resistance anisotropy at room temperature ($R_{out\text{-}of\text{-}plane}/R_{in\text{-}plane}$) was increased by $\sim$5 times (1.48 to 7.19) during endotaxial synthesis. In-plane measurements, both 2-wire and 4-wire, were performed at room temperature in all permutations between bottom and top contacts. 2-wire measurements between (T2, T3) and (B4, B5, B6) with known contact resistances were used to derive a mean out-of-plane resistance. Out-of-plane to in-plane resistance ratio defines the resistance anisotropy. Contact resistances were extracted from in-plane measurements. TaS_2 was measured before and after endotaxial conversion into a mixed polytype with 2D CDWs.

	Pre-treatment	Post-treatment
Anisotropy (Out-of-plane/In-plane)	1.48	7.19
Resistance (Ω)		
In-plane	12.48	5.84
Out-of-plane	18.45	42.01

Supplementary Table 1 | Resistance Anisotropy Measurements in Pristine and Endotaxial TaS_2

Supplementary Note 6 Electronic Device Overview

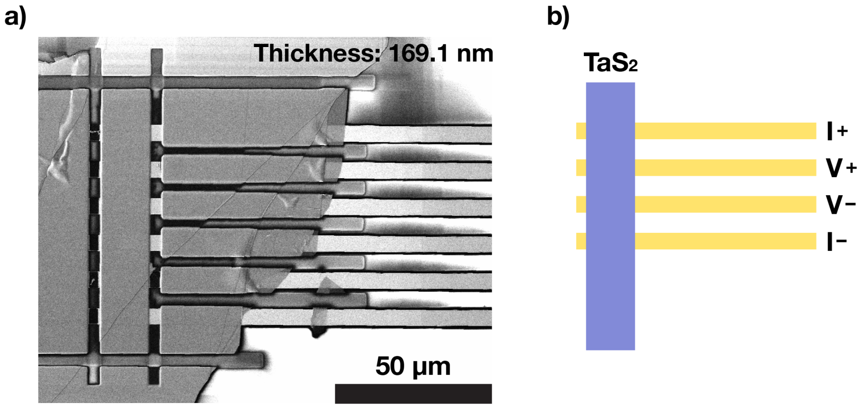

Supplementary Figure 6 | Electronic Device Overview. a) Scanning electron microscopy (SEM) image and b) schematic diagram of TaS_2 device. A flake of TaS_2 is transferred on to pre-patterned gold electrodes. The flake then was sculpted to a rectangular bar shape, using focused ion beam (FIB). The sample thickness was measured by atomic force microscopy (AFM).

Supplementary Note 7   Thermal Evolution of oIC-CDW Superlattice Peaks

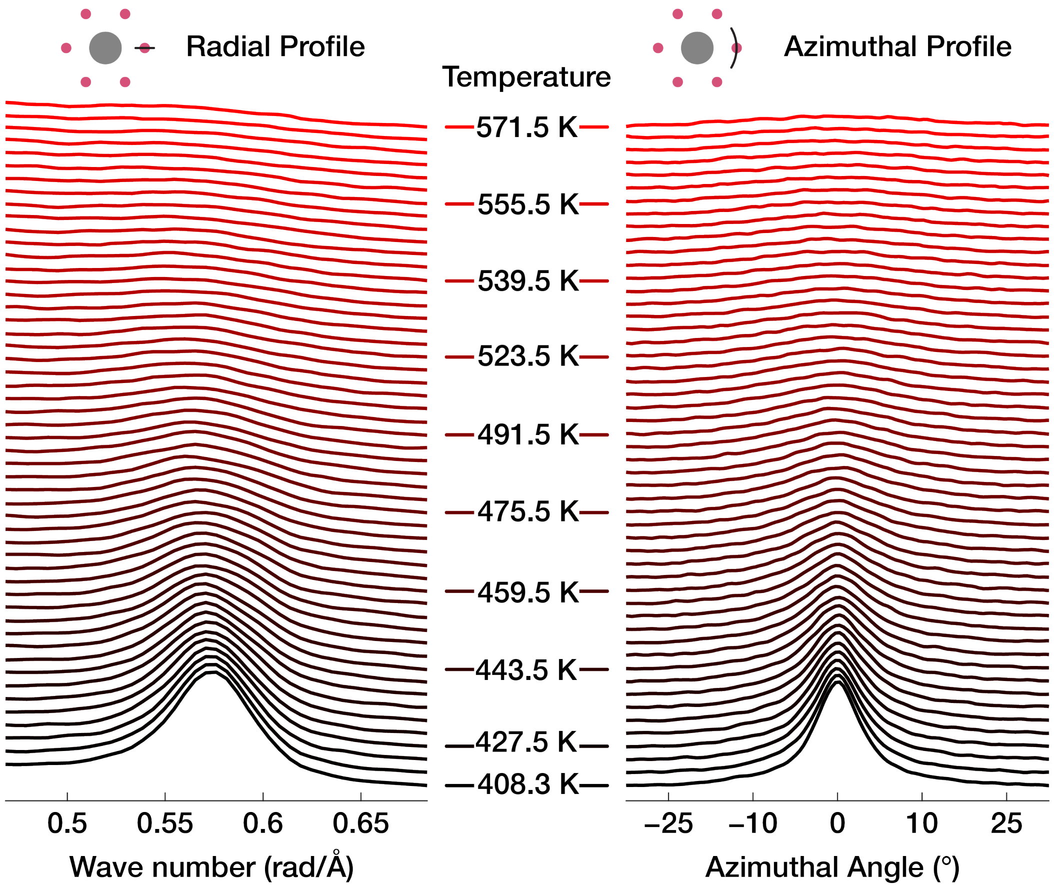

Supplementary Figure 7 | Thermal Evolution of oIC-CDW Superlattice Peaks. Intensity profile of superlattice peak in oIC-CDW phase along a) radial and b) azimuthal direction as temperature increases from 408.3 K (bottom, black) to 571.5 K (top, red). Continuous peak broadening and amplitude reduction is apparent.

Supplementary Note 8 Simulated Hexatic Melting of Charge Lattice and Associated Lattice Distortion

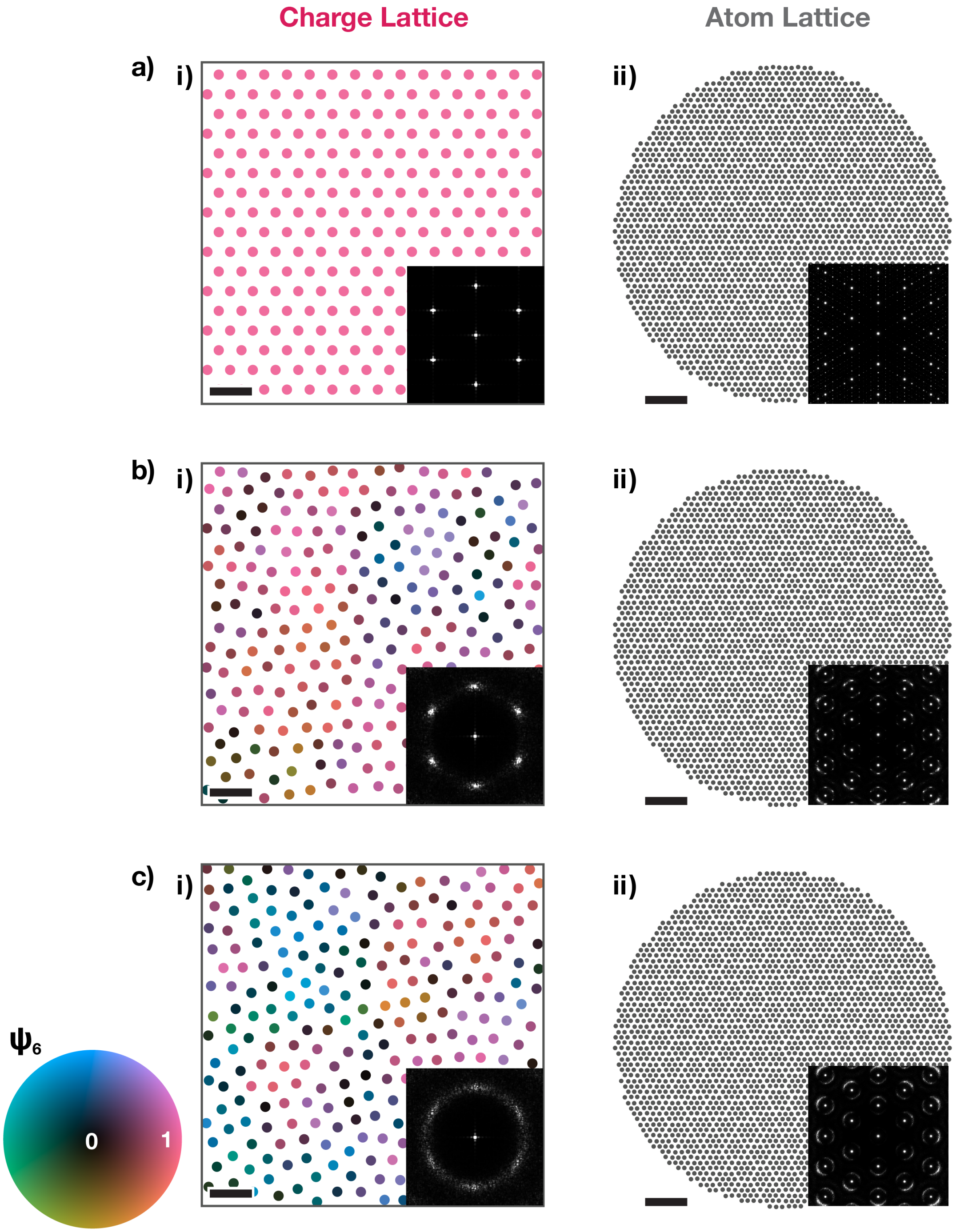

Supplementary Figure 8 | Simulated Hexatic Melting of Charge Lattice and Associated Atomic Lattice Distortion. a-i) Pristine hexagonal charge lattice without disorder is presented. Structure factor (inset) shows sharp well defined six-fold symmetric peaks. a-ii) charge lattice attracts nearby atomic lattice sites forms clusters of atoms. Here, the charge and atomic lattices are incommensurate and each cluster is distinct. The clustering manifests as superlattice peaks decorating Bragg peaks. b-i) The charge lattice is thermally disordered—simulated by a Monte Carlo method (Metropolis-Hastings algorithm) using Boltzmann statistics and truncated Lennard-Jones potential for interparticle interactions. In 2D, crystal can melts hexatically and structure factor (inset) features characteristic azimuthally blurred peaks. c-i) Simulating with increased temperature further disrupts the charge lattice. Structure factor (inset) blurs azimuthally strongly. b, c–ii) Associated lattice distortion with disordered charge lattice is seemingly indistinguishable, however the structure factor (inset) shows azimuthally diffused superlattice peaks. Notably, the superlattice peak structure around each Bragg peak strongly resembles the structure factor of the charge lattice. Scale bars are 20 Å. Left-bottom) The magnitude and the argument of 6-fold bond order parameter ψ_6 is mapped to saturation and hue of the color wheel. The magnitude ($|\psi_6|$) and the argument ($\mathrm{Arg}[\psi_6]$) represents a amount of 6-fold orientational order and local orientation at each lattice site, respectively.

Supplementary Note 9 Comparison of Charge Lattice Model and Experimental Diffraction Patterns

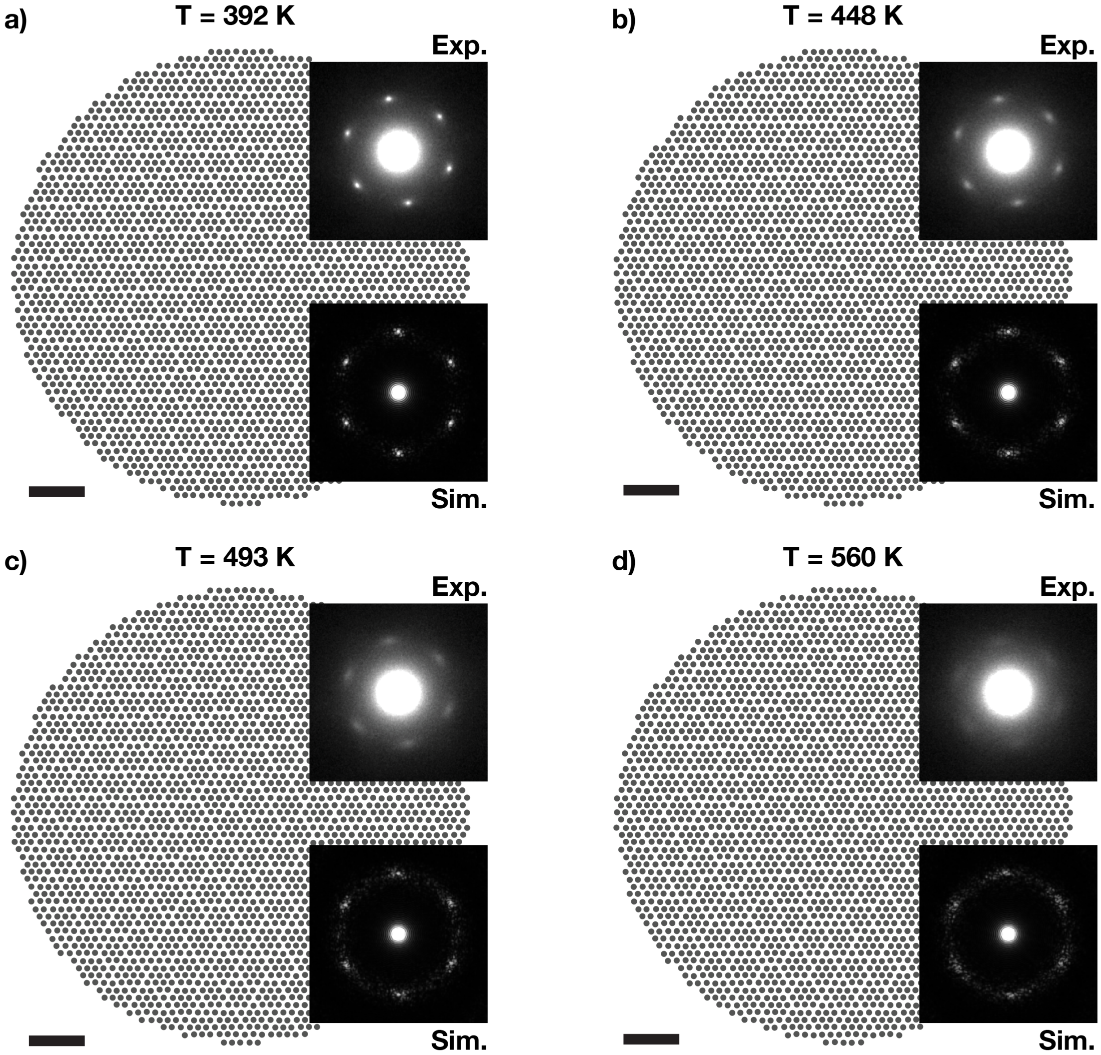

Supplementary Figure 9 | Comparison of Charge Lattice Model and Experimental Diffraction Patterns. a–d) Increase in temperature (T = 392 K, 448 K, 493 K, and 560 K) disrupts CDW order, here simulated with hexatically melting charge lattice. Atomic lattice with associated lattice distortion displayed. The hexatic disorder azimuthally blurs the superlattice peaks. Simulated (inset-bottom) and experimental (inset-top) directionally averaged diffraction patterns qualitatively match. Scale bars are 20 Å.

Supplementary References

[1] J. Wilson, F. Di Salvo, and S. Mahajan, "Charge-density waves and superlattices in the metallic layered transition metal dichalcogenides", Adv. Phys. **24**, 117–201 (1975).

[2] A. W. Overhauser, "Observability of charge-density waves by neutron diffraction", Phys. Rev. B **3**, 3173–3182 (1971).

[3] R. Hovden, P. Liu, N. Schnitzer, A. W. Tsen, Y. Liu, W. Lu, Y. P. Sun, and L. F. Kourkoutis, "Thickness and stacking sequence determination of exfoliated dichalocogenides (1t-tas$_2$, 2h-mos$_2$) using scanning transmission electron microscopy", Microsc. Microanal. **24**, 387–395 (2018).

[4] I. El Baggari, B. H. Savitzky, A. S. Admasu, J. Kim, S.-W. Cheong, R. Hovden, and L. F. Kourkoutis, "Nature and evolution of incommensurate charge order in manganites visualized with cryogenic scanning transmission electron microscopy", Proc. Natl. Acad. Sci. **115**, 1445–1450 (2018).

[5] S. H. Sung, Y. M. Goh, H. Yoo, R. Engelke, H. Xie, K. Zhang, Z. Li, A. Ye, P. B. Deotare, E. B. Tadmor, A. J. Mannix, J. Park, L. Zhao, and R. Kim P. Hovden, "Two-dimensional charge order stabilized in clean polytype heterostructures", Nat. Commun. **13**, 7826 (2022).
