# Supplementary material for: Endotaxial stabilization of 2D charge density waves with long-range order
Source: Nat Commun. 2024 Feb 15;15:1403. doi: 10.1038/s41467-024-45711-3 (PMC10869719; doi:10.1038/s41467-024-45711-3)
Supplement: Supplementary file 3 — Description of Additional Supplementary Files [file 41467_2024_45711_MOESM3_ESM.pdf]

File Name: Supplementary Information.pdf
Description: **Supplementary Information for Endotaxial Stabilization of 2D Charge Density Waves with Long-range Order**

File Name: Supplementary Movie 1.mp4
Description: **In-situ TEM movie of layer-by-layer Octahedral to Prismatic polytype transformation**
